# Densification, Microstructure, and Mechanical Properties of Additively Manufactured 2124 Al–Cu Alloy by Selective Laser Melting

**DOI:** 10.3390/ma13194423

**Published:** 2020-10-05

**Authors:** Junwang Deng, Chao Chen, Wei Zhang, Yunping Li, Ruidi Li, Kechao Zhou

**Affiliations:** State Key Laboratory of Powder Metallurgy, Central South University, Changsha 410083, China; JWDeng001@outlook.com (J.D.); lyping@csu.edu.cn (Y.L.); liruidi@csu.edu.cn (R.L.); zhoukechao@mail.csu.edu.cn (K.Z.)

**Keywords:** additive manufacturing, selective laser melting, Al–Cu alloy, mechanical properties, microstructure

## Abstract

Owing to its high specific strength and low density, Al–Cu alloys have been extensively used in aerospace for lightweight components. Additive manufacturing techniques such as selective laser melting, which offers geometric freedom, is suitable for topology-optimized designs. In this study, the effect of processing parameters on the densification, microstructure, and mechanical properties of additively manufactured Al–Cu alloy 2124 by selective laser melting was investigated. Parameters such as laser power, scanning speed, hatch spacing, and use of a support were studied. The results revealed that a grille support with a hollow structure played a resistant role in the transfer of heat to the base plate, thus reducing the temperature gradient and lessening cracks in the building part. Smaller hatch spacing was beneficial for the achievement of a higher relative density and strength due to track re-melting and liquid phase backflow, which could fill cracks and pores during the building process. An ultimate tensile strength as high as 300 MPa of the vertically built sample was obtained at optimized processing parameters, while the elongation was relatively limited. Moreover, columnar grains were found to be responsible for the anisotropy of the mechanical properties of the as-printed 2124 alloy.

## 1. Introduction

Additive manufacturing (AM) is an advanced technique characterized by a layer-by-layer fabrication process that produces parts with a complex geometry [1]. As one of the most important AM techniques, selective laser melting (SLM) has attracted extensive attention in both research and industrial fields. On the other side, aluminum alloys have been extensively used in aerospace, spaceflight, military equipment, shipbuilding, automotive industry, and electronic engineering owing to their low density, comparatively high strength, and high corrosion resistance, etc. [2]. Due to the great demand for AM parts made from Al alloys, SLM processing of Al alloys such as Al–Si [3,4], Al–Zn [5], Al–Cu [6,7], Al–Sc [8], and Al–Mg–Sc–Zr [9] have been studied systematically. The concept of adjusting material composition to fit the AM process was proposed to meet more application requirements [10,11]. However, due to the inherently high laser reflectivity and high thermal conductivity of Al alloy powders, it is generally difficult to produce Al alloy parts with a high performance by SLM. It means that producing Al alloy parts needs a higher energy density, which is easy to cause pores, cracks, inclusions and other defects in parts because of metal splashing and greater residual stress during printing. These defects will reduce the performance of parts.

Al–Cu alloys are the most widely used Al alloys, accounting for 45% of the Al alloys used in civil aircrafts because of their higher strength compared to other Al alloys [12]. Al–Cu alloys play an important role in reducing weight, saving energy, and decreasing costs in the aerospace, military equipment, shipbuilding, and automotive industries. Nevertheless, due to the previously mentioned problems [4] of Al–Cu alloys, SLM of Al–Cu alloys is much more complex and difficult compared to that of Al–Si alloys. The typical defects observed in Al alloys are porosity, residual stress, cracks, and shape distortion during the building process [13]. The minimization of these defects remains an important topic for the SLM of Al alloys. In general, there are two approaches to reduce or eliminate defects. One is to optimize the process parameters, and the other is to adjust the alloy composition [14]. Process parameters such as laser power, scan speed, hatching space, scanning strategy, and platform temperature can strongly affect the formation of defects. These are great significance regarding how to reduce the defects of cracks, pores and others to popularize the application of Al–Cu alloys in SLM. The effects of scanning speed, laser power, hatching space, building direction and support on material properties, cracks and pores were systematically studied, which could provide a good reference value for the subsequent research on Al–Cu alloy AM.

The 2124 alloy is the most widely used Al–Cu alloy for aircraft frames and components such as fuselage, wing skin structure, wing beam, wing rib, and partition frame. It is characterized by a high strength-to-weight ratio, specific stiffness, fracture toughness, and good corrosion resistance [15]. Therefore, the 2124 alloy was selected as the research subject in this study. In this work, the feasibility of an SLM-manufactured 2124 Al–Cu alloy was explored by varying the process parameters, including laser power, scanning speed and hatching space. Moreover, the effect of support and process parameters on orientation, phase transition and solidification behavior during the SLM process was investigated to improve the performance of Al–Cu alloy parts.

## 2. Experimental

### 2.1. Powder Material

**Figure 1:**
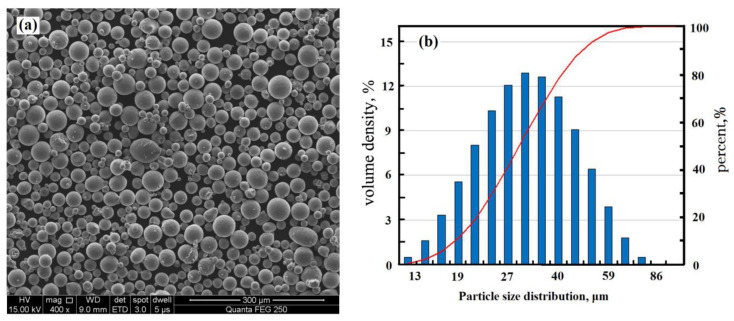
(**a**) SEM images of the powders and (**b**) the corresponding particle size distribution.

Nearly spherical powder was produced by gas atomization. As shown in Figure 1, the particle size distribution detected by the laser diffraction particle size analyzer (Malvern Micro-plus, West Midlands, UK) was in the range of 13 to 76 μm, with a median particle size (D50) of 33.8 μm. The detailed chemical composition of the 2124 alloy is shown in Table 1. Before the SLM process, the powder was dried by drying ovens at a temperature of 393 K to reduce the humidity. According to previous studies, drying the powder before AM can reduce porosity by 50% and greatly improve the qualification of the AM parts [16,17].

### 2.2. SLM Method and Processing Parameters

**Figure 2:**
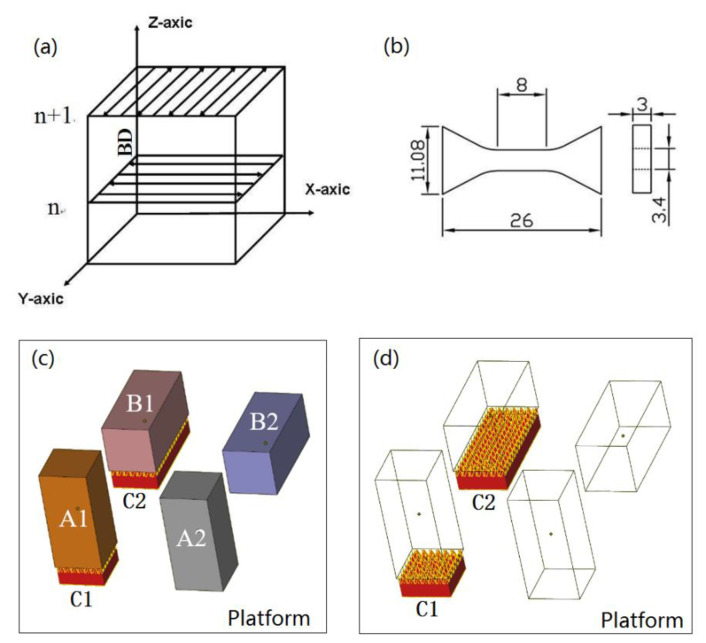
(**a**) Schematic diagram of scanning strategy. (**b**) The test sample schematic after machining from middle part of the SLM sample; the units are mm. (**c**) The orientation of the building specimens, A1 is the vertical sample with support and A2 is without support, B1 is the horizontal sample with support and B2 is without support. The size of A1, A2, B1 and B2 is 12 × 12 × 30 mm. (**d**) C1 and C2 are supports under A1 and B1. The height of C1 and C2 is 3 mm.

The SLM process was carried out in a FS271 SLM machine equipped with a Gaussian beam fiber laser. The maximal power was 500 W, and the laser beam diameter was 100 μm. A scanning strategy with rotation of 90°, as shown in Figure 2a, was used to build the samples. To investigate the influence of different building orientations on the properties of the samples [18], vertical and parallel orientations of the building specimens with or without supports were studied, as shown in Figure 2c. The longitudinal samples denoted as group A were parallel to the building direction. The transverse samples referred to as group B were perpendicular to the building direction. Group C was the support of the building samples. Diamond supports were used in this study, as shown in Figure 2d. Table 2 shows the manufacturing parameters of the SLM used in this work, including laser power, laser scanning speed, hatching space, layer thickness, and temperature of the platform.

### 2.3. Sample Characterization

Archimedes’ law was used to test the density three times per SLM sample and calculate the average relative density of two samples. Light microscopy (LM, MeF3A, Leica, Germany) and scanning electron microscopy (SEM, JSM-6360LV, JEOL, Tokyo, Japan) with a secondary electron detector were used to examine the microstructures of the samples. The sample was set at room temperature with denture powder and solvent, and then polishing the sample via water-cooled metallography, the prototype was set to get the metallography sample. Before observation by LM, the samples were etched with Keller’s reagent. The mechanical properties of the samples with the geometry shown in Figure 2b were tested using an Instron 3369 mechanical testing machine at room temperature, and the speed rate in the static tensile tests was 1 mm/s. The mechanical properties of the samples were the average value of two samples. The microhardness was measured using a micro-Vickers hardness machine (600HVS-1000AVT, Huayin Test Instrument Co., Ltd., Hunan, China) under a load of 100 g for 15 s based on three randomly chosen points from the middle region of one part melted by SLM and the average value of two parts. Samples were prepared on RL-I twin-jet electro-polishing device for 20 s at 30 V in corrosive liquid at −35 ℃, which used for the electron backscatter diffraction (EBSD) observation with a scanning step of 0.2 μm. The machine of EBSD is FEI, HELIOS Nano Lab 600i. The energy disperse spectroscopy (EDS) mapping analysis was conducted in the JSM-6360LV SEM to study the element distribution in samples melted by SLM.

## 3. Results

### 3.1. Optimization of Laser Power and Scanning Speed

**Figure 3:**
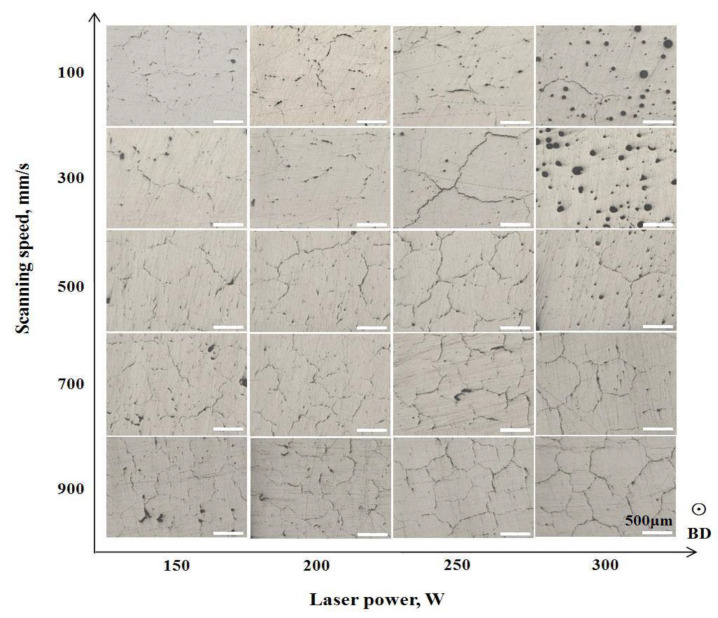
The microstructure of SLM-printed horizontal samples without supports at various laser powers and scanning speeds.

Both laser power and scanning speed are the most important parameters in the SLM process. To investigate the effect of these two parameters on the building quality of the samples, the laser power was set to 150, 200, 250, or 300 W, and the scanning speed was set to 100, 300, 500, 700, or 900 mm/s. Figure 3 shows the embedded light micrographs of the SLM samples under various laser power and scanning speed parameters. Two main defects, including pores and cracks, were observed in the microstructures. There was a large number of pores in the samples at the higher power of 300 W or lower scanning speeds of 100 and 300 mm/s. With increased scanning speed, the number of pores decreased and were replaced by a large number of cracks. More cracks were generated at higher scanning speeds. The aforementioned results indicated that neither a high laser power nor fast scanning speed was suitable for SLM in the present condition. In this study, the most suitable parameters for the SLM building of Al alloy 2124 were determined to be a laser power of 150 W and a scanning speed of 100 mm/s.

### 3.2. Effect of Supports

**Figure 4:**
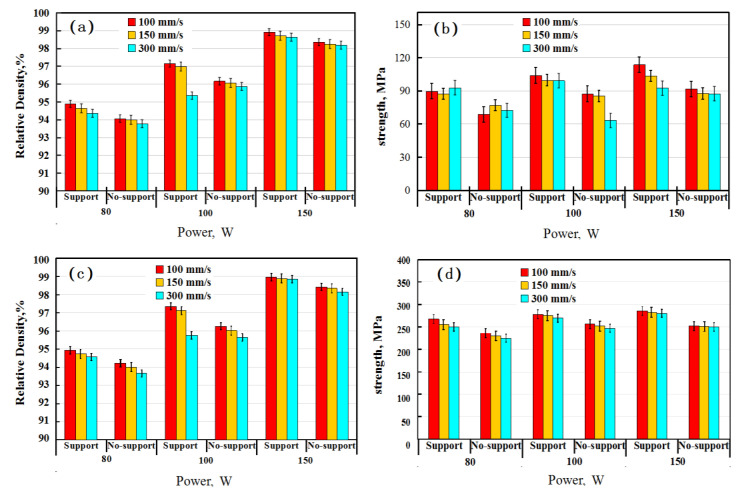
Effects of power and scanning speed on the relative density and strength of horizontal (**a**,**b**) and vertical (**c**,**d**) samples produced with and without support.

Supports are widely used in the SLM process to avoid the distortion and/or cracking of the building parts. With a support below, a part can enhance the temperature uniformity during the building process due to heat transfer and also reduce the warping deformation of the forming part by maintaining the stress balance [19]. The use of a support is also beneficial for improving the density and mechanical properties of building parts [20,21]. The effects of power and scanning speed on the relative density and strength of samples with or without support are shown in Figure 4. It is clear that the relative density and strength of the horizontal and vertical samples with support were both higher than those of the samples without support. It has been reported that the bottom support can reduce the stress values of the printed sample [22]. Thus, it could inhibit the formation of cracks due to residual stress debasing during processing.

**Figure 5:**
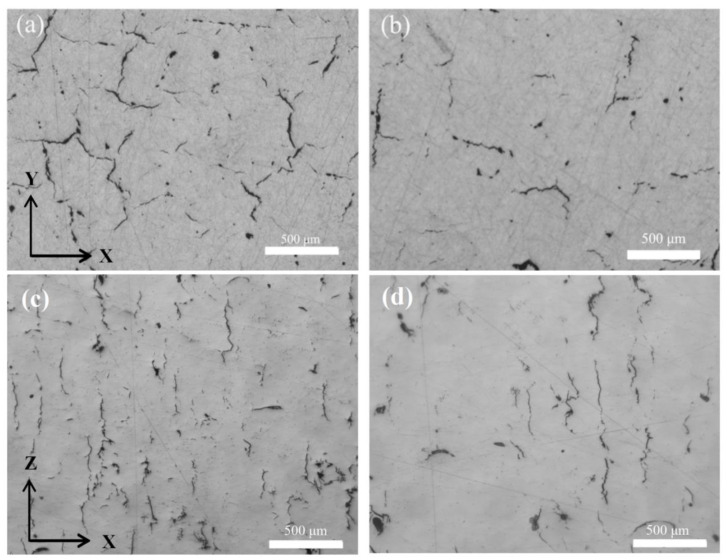
Light microscopy (LM) images showing the morphology of samples without (**a**,**c**) and with (**b**,**d**) support. These images showing two types of crack distribution—transverse cracks distributed sporadically in the sample cross-section and longitudinal cracks parallel to building direction.

Figure 5 shows the LM images of the samples without and with support. As can be seen, the cracks in the images presented in Figure 5a,c are numerous and some are interconnected, while the number of cracks is scattered and few are in image Figure 5b,d. This object is supported by a grille support with a hollow structure. It can increase the resistance and time of thermal transfer from the sample to the base plate, which results in more residual heat in the sample compared with the block structure. The rate of heat transfer becomes slower, which causes more heat and reduces the rate of heat loss and maintains the sample at a higher (or more stable) temperature during the manufacturing process. The cyclic superposition of residual heat can raise the temperature of the sample, which may reduce the temperature gradient and residual stress of the samples. The relative density and tensile strength of the horizontal and vertical sample with support were 98.91% and 98.95% and 113.03 MPa and 285.78 MPa, respectively. For horizontal and vertical samples without support, the relative density was 98.36% and 98.42%, and the tensile strength was 91.67 MPa, 262.73 MPa. Thus, the support adopted in this study can reduce the generation of cracks, thus leading to the achievement of a higher density and strength.

### 3.3. Effects of Building Direction

**Figure 6:**
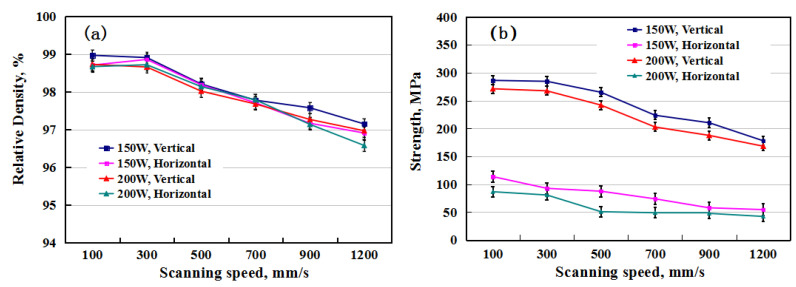
(**a**) The relative density and (**b**) strength changes with scanning speed for the vertical and horizontal samples, respectively.

Figure 6a,b show the relative density and tensile strength of the specimens with vertical and horizontal orientations, respectively. Both the relative density and strength of the specimens decreased with an increasing scanning speed for both vertical and horizontal samples. The properties of the samples produced at 150 W were better than those at 200 W. It is worth noting that at laser powers of 150 and 200 W, the strengths of the vertical samples were much higher than those of the horizontal ones, which showed the anisotropy of the mechanical properties of the samples. The highest relative density of the sample at vertical ordination reached 98.97%, and the corresponding tensile strength was the highest at 286 MPa at 150 W and 100 mm/s. However, under the same SLM conditions, the strength of the horizontal sample was only 113 MPa with a similar relative density. In comparison, the strength of the 2124 alloy was 180 MPa by conventional casting [23]. This demonstrates that the tensile strength of the vertical sample by SLM was higher than that of the casting counterpart, while the horizontal samples showed much lower tensile strengths. The anisotropy of mechanical properties also exists in other Al alloys, such as the SLM 7075 Al alloy [24]. According to the data in Figure 4 and Figure 6, the tensile performance of the vertical sample was much better than that of the horizontally printed sample, and subsequent studies mainly focus on the vertical sample.

### 3.4. Effect of Hatching Space

**Figure 7:**
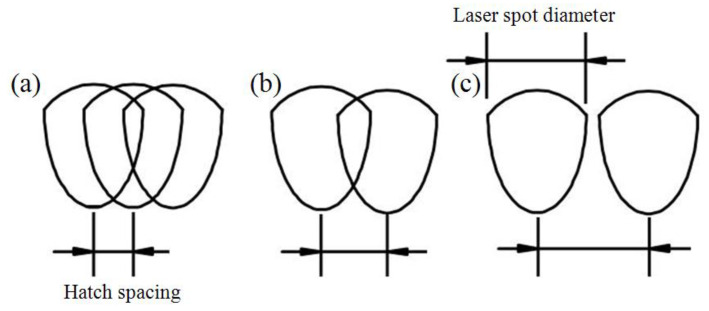
SLM build-up from overlapping of hatch spacing. (**a**) More and multiple overlap, (**b**) normal overlap, (**c**) no overlap.

Hatch spacing is an important parameter for the SLM process. From Equation (1), the energy density decreases with increasing scan spacing. Simultaneously, various scan hatch spacings will produce different overlap areas, as shown in Figure 7. A small hatching space will lead to a larger overlap area and multiple overlaps, while a large distance of hatch scanning may cause adjacent weld tracks without overlapping due to the large gap. A normal overlap of weld tracks is needed to produce parts of high quality in SLM, as shown in Figure 7b. So far, no previous research has specifically determined what area of overlap is the most appropriate for building parts. This is a complex issue, because the overlap area is associated with many factors such as hatch spacing, laser energy density, fluidity of the melting pool, and the formation of oxide film during the printing process [25,26].

**Figure 8:**
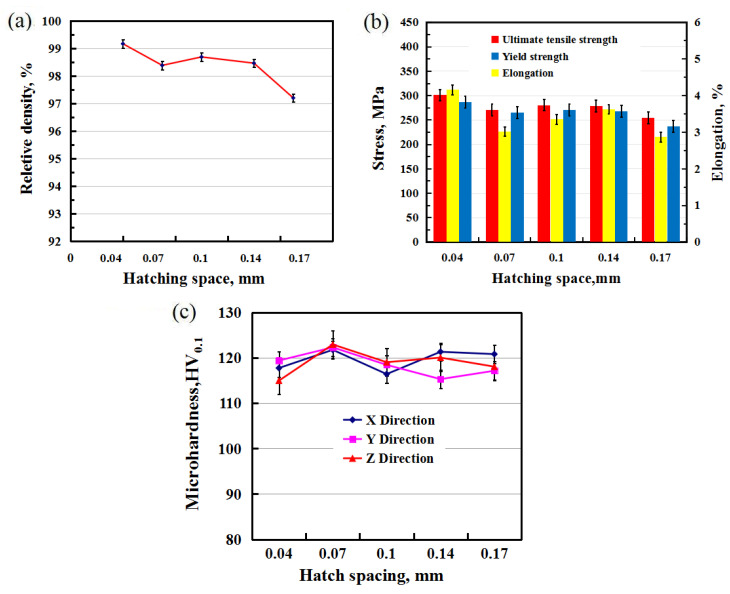
(**a**) Relative density, (**b**) stress, and (**c**) microhardness of SLM-printed 2124 at different hatch spacings of vertical samples with support.

In this study, hatch spacings of 0.04, 0.07, 0.1, 0.14, and 0.17 mm were used to study the influence on the microstructure, density, and mechanical properties of the samples at a power of 150 W and a scanning speed of 100 mm/s. Figure 8 shows the relative density, tensile strength, and microhardness of SLM-manufactured 2124 at different hatch spacings. Both the density and strength of the samples reduced as the hatch spacing increased, as shown in Figure 8. At a hatching space of 0.04 mm, the highest relative density and ultimate tensile strength of the sample reached 99.17% and 300.96 MPa, respectively. The relative density was 97.2%, and the ultimate tensile strength was 254.26 MPa at a hatch spacing of 0.17 mm. The values were very similar for the remaining hatch spacings of 0.07, 0.1, and 0.14 mm. However, the elongations of all samples were very low because of pore and crack defects, with the cracks being the main reason for the brittle fracture during testing of SLM 2124. It is worth noting that the microhardness of the samples showed little difference for all the hatch spacings tested, even in the X, Y, and Z directions, as shown in Figure 8c. Each value represents the average microhardness of three measurements. Microhardness is mostly determined by the microstructure [27]. The microhardness was low at the pore or crack edges and high in the alloy matrix, which made it difficult to determine the true microhardness value of the material.

**Figure 9:**
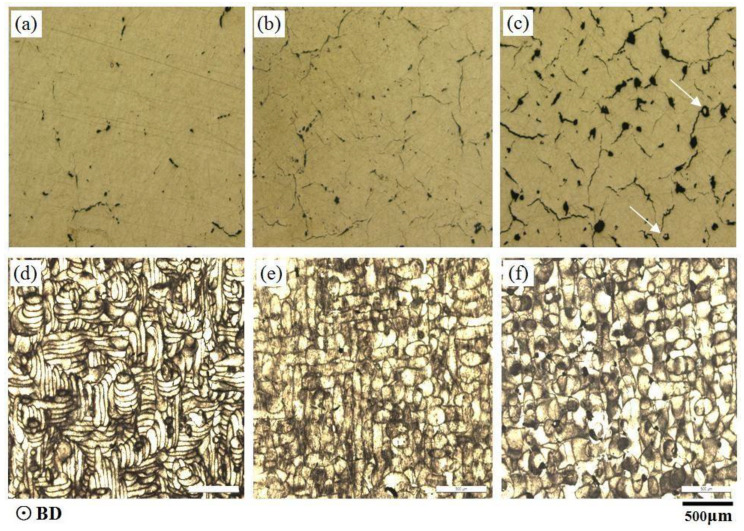
LM images show the etched morphologies of the SLM 2124 transversal surface at hatch spacings of (**a**,**d**) 0.04, (**b**,**e**) 0.1 and (**c**,**f**) 0.17 mm.

Figure 9 shows LM images at hatch spacings of 0.04, 0.1, and 0.17 mm, respectively. There were fewer porosities and cracks in the 0.04 mm sample, characteristic of the narrower scanning tracks at this hatch spacing, and the corresponding tensile strength was 300.96 MPa. With increased hatch spacing, the pores became more frequent and larger, the cracks became longer, and some cracks were connected to each other. These increased defects coincided with the wider tracks and lower strength of 254.26 MPa at a hatch spacing of 0.17 mm. In addition to the pore and crack defects, non-molten powders were enclosed in the sample, as shown in Figure 9c, which were mainly attributed to the non-overlap between tracks due to an excessive hatch spacing of 0.17 mm. It can be seen from this study that a smaller hatch spacing with greater overlap can improve the properties of SLM-printed parts. More overlap means more re-melting during the process, which is conducive to obtaining fewer defects, as well as denser and higher-strength parts.

## 4. Discussion

Porosity and cracks were the most frequently observed defects in the SLM parts, as shown in Figure 3 and Figure 9. The small-size pores were approximately several microns in size, while the large pores were tens of microns and some larger pores were more than a hundred microns. The shapes of the pores were also different: one was a spherical morphology and the other was an irregular morphology. The formation of pores with various sizes and shapes was attributed to their distinctive formation mechanisms, which could also be classified into keyhole pores with a larger size and irregular shape, and metallurgical pores with a smaller size and spherical shape [28]. The slower the scanning speed, the more keyhole pores were formed during SLM processing at a certain laser power (300 W) shown in Figure 3, contrary to the results of the previous study that found that the pores increased with scanning speed [29]. This may be associated with melting pool instabilities due to the laser spatter, which could form small droplets of material that splashed out from the pool [30]. The production of the spatter is related to the energy density of the input laser, which can be determined by the equation for the laser energy density, where *E* is directly proportional to *p* and inversely proportional to *v* [31].

*E* = *p/vht* (1)

where *E* is the energy density of the input laser, *p* is the laser power, *v* is the scanning speed, *h* is the hatch spacing, and *t* is the layer thickness.

It was found that an overly low power leads to incomplete melting. However, a higher *p* produces a higher *E* and higher superheat of the melting pool, but excessive superheat leads to the volatilization of low-melting point metals and spattering during the building process. In addition, a high superheat of the melting pool would easily result in a larger temperature gradient in the sample because SLM is a process of layer-by-layer scanning with cyclic heating. Thus, a somewhat lower *p* could be beneficial as it can not only eliminate volatilization and spatter, but also reduce the temperature gradient without interfering with complete melting.

Considering the other parameters, the smaller the *v* value, the greater the *E* value. A higher energy density can easily create the evaporation of elements that leads to spatter during building [28]. Spattering can cause the surface of SLM layers to be uneven, which creates various pores in the subsequent printing process. The larger the energy density, the more intense the spattering created, and therefore, the greater the number of pores. Slowing the scanning speed can make the energy input of the printing process slower and more uniform, which can decrease the temperature gradient of the part due to the reduction in instantaneous heat impact. Therefore, reducing *v* is beneficial for obtaining higher-quality building parts.

Another main defect are the cracks in the SLM Al alloy. The formation of cracks is mainly determined by the inherent characteristics of the alloy itself. A 2124 Al alloy with about 4% copper is a typical hypoeutectic alloy from the Al–Cu phase diagram, which is a feature with a wide range of crystallization temperatures. The eutectic temperature is 548.2 °C and the precipitated strengthening phase is the θ phase, namely Al_2_Cu, which is a low-melting phase. The low-melting phase usually solidifies last and is easily distributed along the grain boundary. The Al_2_Cu precipitates either disperse in the molten pool in granular, or form dendrites in the molten pool boundary, or form the continuous line-like precipitates in the columnar grain boundary [32]. The solidification stage of subeutectic Al-Cu alloy could be divided into quasi-liquid zone, hot brittle zone and low strength plastic zone [33]. The hot brittle zone had a great tendency for hot cracking during the process of solidification. This zone was characterized by a solid-liquid coexistence and a wide range of solidification temperatures. When the copper content was in this zone, the Al-Cu alloy had a greater tendency of hot cracking during solidification, especially when the copper content ranged from 3% to 5%, which had a very strong tendency for hot cracking [34]. In this study, the copper content was approximately 4% of the alloy, which was just in the hot brittle zone, and it was easy to produce thermal cracks. The cracks were produced by local shrinkage stress when the liquid phase backflow was not sufficient at the end of solidification. Cracks might also develop after the alloy completely solidified, due to thermal cycling. A uniform cooling distribution of the Al-Cu alloy during solidification could greatly diminish and even eliminate these thermal cracks. However, the temperature gradient in samples produced by SLM is relatively large, due to the continuous action of laser scanning and the steep cooling curves with the process, which results in much residual stress. As the scanning speed increases the temperature gradient also increases, which induces more residual stress and produces more cracks. Similar results were found for the SLM 2024 Al-Cu alloy [35].

In the traditional casting of Al–Cu alloys, a lower superheat of the alloy melt and a slower cooling rate are often used to reduce the temperature gradient during solidification for eliminating hot cracks. Analogously, in this study, an appropriately low laser power means a suitable superheat and a slower scanning speed means a slower input of heat. A grille support with a hollow structure is responsible for the heat transfer to the base plate. The superposition of these parameters can help to reduce cracks because they can maintain a certain amount of heat and slow down the cooling rate, which may reduce the temperature gradient and residual stress of the building part.

A small scanning space can increase the overlap of the track and the melting time. The re-melting of the track caused the liquid phase to redistribute and Al_2_Cu, the precipitated phase with a low melting point, to fill the cracks and pores. In this process, the t value is only 30 μm, which is the distance between the building layer and the scanned layer. It is so short that the laser can penetrate the building layer and act on the under layer by liquid phase backflow. The greater the re-melting time, the better the effect, which can be conducive to reducing cracks and pores.

**Figure 10:**
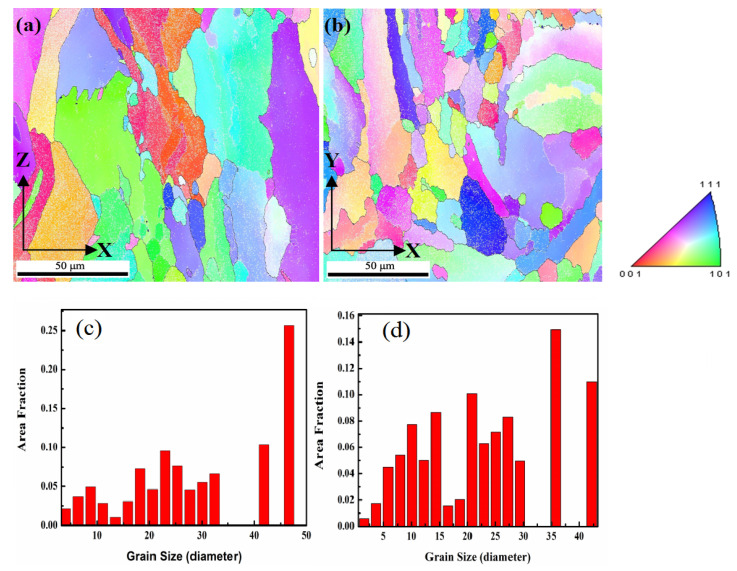
Electron backscatter diffraction (EBSD) micrographs showing the grain orientation and distribution in the SLM samples. (**a**) Map showing columnar crystals in vertical orientation. The white dotted box and arrows indicate relationship between loading direction and grain boundary of the columnar crystal. (**b**) Map showing irregular crystals in horizontal orientation. (**c**) The grain size of produced sample in vertical orientation. (**d**) The grain size of produced sample in horizontal orientation.

The SLM process has an intrinsic characteristic of extremely high cooling rates of ~105–106 K/s, which can substantially refine the microstructure in produced samples [36]. The range of grain sizes was from about 1 to 50 μm both in vertical and horizontal orientation-produced samples, as shown in Figure 10c,d. The substantially refined grian was caused by the precipitation of a large number of low melting point Al_2_Cu during production due to the extremely high cold rate. It explained why the tensile properties of samples melted by SLM in the vertical orientation were much higher than those of traditional 2124 Al–Cu alloys. This phenomenon had been confirmed by a previous study, and the precipitation was Al_2_Cu [32]. Microstructure refinement can greatly improve the mechanical properties of aluminum alloy samples produced by SLM, which has also been demonstrated by previous studies [37]. The microstructure formation is controlled by the thermal history during processing, which suffers from heat transfer and thermal gradients. Along the building direction in the samples, the main morphology was columnar crystals in the vertical orientation due to thermal gradients distributed between the higher temperature at the top to the lower temperature at the bottom of the sample, while the horizontally printed samples contained equiaxed crystals, as shown in Figure 10a,b, respectively. The appearance of columnar crystals played a significant role in the tensile strength of the materials in the vertical orientation.

The tensile orientation of the horizontally built samples is perpendicular to the grain boundary of the columnar crystal, while those of the vertically built samples is parallel to the grain boundary, as shown by the white dotted box and arrows in Figure 10a. One loading direction was perpendicular to the grain boundary of the columnar crystal in the vertical sample, while the other loading direction was parallel to the grain boundary of the columnar crystal in the horizontal sample. While the property anisotropy of the SLM sample was attributed to the columnar crystal, there was not a significant difference in the tensile strength value between the horizontal and vertical processing orientations in the crack-free sample [35]. During metal SLM processing, cracks tended to grow along the grain boundary of the columnar crystal, which greatly reduced the mechanical properties of the horizontal sample.

Other major factors affecting the performance anisotropy of the as-fabricated samples were the crack distribution and the tensile direction. There were shrinkage cracks, oxidation cracks, etc., besides the stress cracks. Shrinkage cracks could be attributed to the volume during solidification process which cannot be backfilled in time by melt reflux. The same phenomenon of shrinkage cracks was confirmed in the AlCu5MnCdVA alloy melted by SLM [38]. During solidification in the sample production process, hot cracks formed and grew along the columnar grain boundaries [39], which are also called longitudinal cracks and are parallel to the building direction, as shown in Figure 5c,d. The transverse cracks were seen as the cross section of longitudinal cracks, as shown in Figure 5a,c. According to the solidification phase diagram of the Al–Cu alloy, due to the shrinkage in the solidification brittleness area, the shrinkage stress values are higher than the strength of the semi-solid metal, which leads to thermal cracks along the grain boundaries.

**Figure 11:**
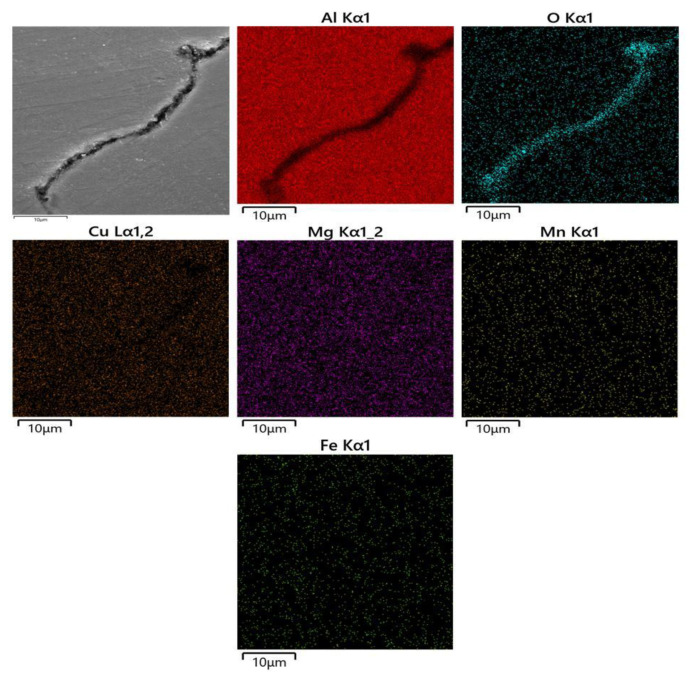
Energy disperse spectroscopy (EDS) mapping analysis results for a crack of Al-rich oxide clusters.

Oxidation cracks were another common form of cracks. Aluminum readily reacted with oxygen to form Al_2_O_3_ due to it being highly active, which should be avoided during SLM. The oxygen content of the protective atmosphere was reduced by inputting high-purity argon. Nevertheless, trace amounts of oxygen could inevitably react with highly active elements such as aluminum. Some of the oxygen reacted with the element of splattering particles generated during laser scanning and were carried away by the flowing atmosphere. The others reacted with active ingredients of the building samples to produce the oxide ceramic phase with poor wettability, which might cause cracks [14]. Figure 11 shows EDS mapping analysis results for a crack of Al-rich oxide clusters. According to the analysis results, there was an oxidation phenomenon around the crack. There was poor Al with red and rich O with green, while there was a uniform distribution of other elements. It meant that aluminum reacted with oxygen, which was preferred over other elements during the building process, suggesting that they should be Al_2_O_3_ [3]. Therefore, oxidation contributed to the formation of cracks.

**Figure 12:**
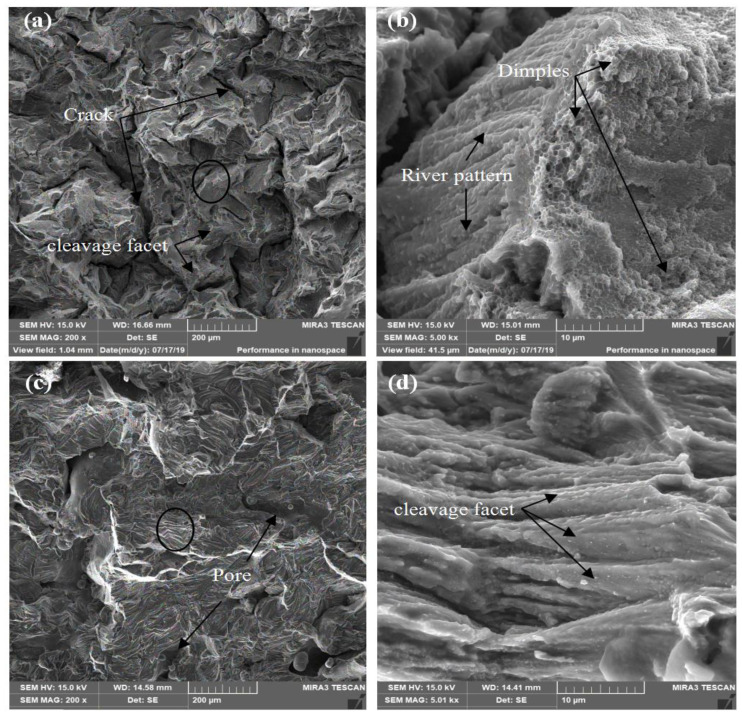
SEM images of the fracture surfaces of SLM-produced 2124. (**a**) Vertically built sample and (**c**) horizontally built sample at room temperature. (**b**,**d**) are enlarged images of the regions in the black circles in (**a**,**c**), respectively.

Figure 12 shows SEM micrographs of the fracture surfaces of the as-produced Al alloy at room temperature. These were different from the fracture surfaces of the vertical and horizontal samples. The defects of the cracks and pores could be observed on the fracture surfaces of the vertical sample in Figure 12a and the horizontal sample in Figure 12c. The fracture surfaces show predominant cleavage facets in both the vertical and horizontal samples. Some dimples can be seen in Figure 12b, which indicates that the properties of the vertically processed sample have some ductile fractures. This can be seen in the vertical sample elongation of 3.8%, which was higher than that of the horizontal sample, which was 2.1% at 150 W and 100 mm/s. During the tensile testing process, the cracks and pores in the sample were the first to be subjected to the expansion force. The cracks in the vertical sample were parallel to the stress direction, so the pores and strength of the sample played the leading roles. After the pores fractured, pits were formed. The fracture surface showed clearly visible cracks, and at the edge of the cracks were river patterns, accompanied by a certain number of dimples, as shown in Figure 12a,b. The testing results determined that the strength was higher than 300 MPa. In the horizontal sample, the residual cracks were perpendicular to the loading direction. The sample was easily cleaved owing to the rapid expansion of the cracks under the effect of shear force, leaving dominant cleavage planes, as shown in Figure 12c,d. Almost no dimples were apparent on these fracture surfaces. The highest strength value measured was 113 MPa, which is much lower than that of the vertical sample.

## 5. Conclusions

This work investigated the microstructure and mechanical properties of Al alloy 2124 samples printed by SLM at various laser power, scanning speed, and hatch spacing parameters. The conclusions are as follows:

- The relative density and strength were reduced with an increased scanning speed, and a higher scanning speed produced more pores or cracks. A slower scanning speed, lower laser power, and use of a support were beneficial for obtaining better sample properties, because they could help reduce the temperature gradient, which was more suitable for the 2124 alloy fabricated by SLM in this study.
- The anisotropic mechanical properties of the samples were investigated, and the properties of the vertical samples were much higher because of the columnar crystals and cracks parallel to the loading orientation. Lower hatch spacing resulted in better mechanical properties, because the denser samples had fewer pores and cracks due to the track re-melting and liquid-phase backflow.
- The highest relative density and ultimate tensile strength of the vertical sample were 99.17% and 300.96 MPa at a power of 150 W and scanning speed of 100 mm/s. However, the elongation was very low, demonstrating a typical brittle material because of the defect in the distribution of pores and cracks.
- Supports used during the 2124 alloy building could reduce the generation of cracks, which could help to obtain samples with higher density and mechanical properties.

Obtaining a high performance 2124 alloy by SLM without cracks and with fewer pores will be undertaken in future work. Apart from optimizing the process parameters, adjusting the composition of alloy 2124 may be a more effective method for improving current printing equipment and experimental conditions.

## Figures and Tables

**Table 1:** The main chemical compositions of the powder used in selective laser melting (SLM) determined by inductively coupled plasma-atomic emission spectrometry.

Element (wt.%)	Cu	Mg	Mn	Fe	Si	Ni	Zn	Ti	Al
Powder	4.10	1.46	0.66	0.38	0.08	0.03	0.03	0.01	Balance
SLM sample	4.16	1.30	0.65	0.41	0.06	0.02	0.02	0.01	Balance

**Table 2:** The manufacturing parameters of SLM used in this study.

Manufacturing Parameter	Value
Laser power *P* (W)	80–300
Scanning speed *v* (mm/s)	50–1200
Layer thickness *t* (μm)	30
Hatching space *h* (mm)	0.04–0.17
Temperature of platform *T* (°C)	180
